# Electrosynthesis of electrochemically reduced graphene oxide/polyaniline nanowire/silver nanoflower nanocomposite for development of a highly sensitive electrochemical DNA sensor

**DOI:** 10.1039/d1ra01301g

**Published:** 2021-05-28

**Authors:** Luyen Thi Tran, Hoang Vinh Tran, Hue Thi Minh Dang, Anh Van Nguyen, Thuy Hong Tran, Chinh Dang Huynh

**Affiliations:** Hanoi University of Science and Technology 1st Dai Co Viet Road, Hai Ba Trung District Hanoi Vietnam luyen.tranthi@hust.edu.vn

## Abstract

A novel nanostructured electrode material based on electrochemically reduced graphene oxide/polyaniline nanowires/silver nanoflowers (ERGO/PANi NWs/AgNFs) was fabricated site-specifically onto a Pt microelectrode (0.80 mm^2^ area) using a three-step electrochemical procedure: electrosynthesis of ERGO, electropolymerization of PANi NWs, and electrodeposition of AgNFs. Synergistic and complementary properties of ERGO, PANi NWs and AgNFs, including high electrochemical activity, large surface area, and high biocompatibility, were obtained. Besides, the electrosynthesis method allowed the direct formation of the desired nanomaterial onto the Pt microelectrode, so the adhesion between the sandwich-structured nanocomposite and the electrode surface was also improved. The optimized ERGO/PANi NWs/AgNFs nanocomposite was used for the first time to develop an electrochemical DNA sensor. As a result, the DNA probe immobilization was facilitated and the electrochemical signals of the DNA sensor were enhanced. The detection limit of the DNA sensor was 2.70 × 10^−15^ M. Moreover, potential miniaturization for fabrication of a lab-on-a-chip system, direct detection, high sensitivity, and good specificity are the advantages of the fabricated DNA sensor.

## Abstract 2

A novel nanostructured material based on ERGO/PANi NWs/AgNFs was electrosynthesized on a Pt microelectrode and was used for the first time to develop an electrochemical DNA sensor.

## Graphical abstract

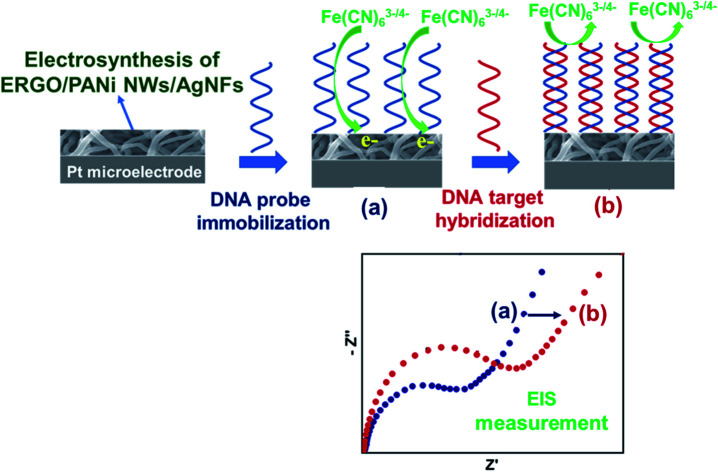

## 1. Introduction

Carbon nanomaterials, including graphene oxide, reduced graphene oxide, graphene and carbon nanotubes, show interesting properties such as large surface area, high electrical/thermal conductivity, good biocompatibility, chemical stability, mechanical strength and cost effectiveness.^1^ The excellent electrochemical characteristics of carbon nanomaterials make them ideal for faradaic and non-faradaic processes. These structures can exchange electrons with adsorbed molecules leading to large changes in conductance.^2^ Due to unique electrochemical and electrocatalytic properties, carbon nanomaterials can be used in various applications including sensors/biosensors, supercapacitors, batteries and fuel cells.^3–6^ For the field of electrochemical biosensors, these materials can help to improve analytical performance.^7^

Nanostructured conducting polymers, especially, polyaniline (PANi) nanostructures have unique characteristics of low-dimensional organic conductors with high surface area, high conductivity, high stability and easy preparation.^8^ The existence of PANi in various oxidation states and protonation degree ranging from the most reduced leucoemeraldine form, through the half-oxidized emeraldine base form to the fully oxidized pernigraniline form, makes it an interesting material.^9^ As a result, nanostructured PANi has a large variety of applications including sensors, nano-electronic devices, catalysts, electron field emitters, actuators, membranes, supercapacitors and batteries.^10–14^ In the field of electrochemical biosensors, PANi is commonly used due to its abilities to act as a linking agent for the immobilization of biomolecules on the electrode surface, and to enhance the efficiency of electron transfer between the transducer and the electrode surface.^15^

Besides, nanostructured metals, especially, nanostructures of silver, such as silver nanoparticles (AgNPs), silver nanowires (AgNWs) and silver nanoflowers (AgNFs), exhibit excellent chemical and physical properties, so they are intensively studied and applied.^15–17^ Thanks to special electrochemical properties, unique catalytic activity, high biocompatibility and reasonable cost, AgNPs, AgNWs and AgNFs are used in the development of electrochemical biosensors to amplify the signal and improve the sensitivity of the sensors.^15,18–20^ In previous studies, AgNPs and AgNWs have been decorated on carbon nanomaterials or conducting polymers to enhance the electrochemical activity of the nanocomposites.^15,18,21,22^ However, there have been no reports on the synthesis of AgNFs embedded in carbon nanomaterials/conducting polymers for fabrication of electrochemical biosensors, which then will broaden the scope of applications of nanocomposites based on silver nanostructures.

The advances in materials research, such as nanocomposites, have promoted their electrochemical applications, including electrochemical DNA sensors.^23^ For the case of carbon/PANi/metal nanocomposite, due to the synergy of the properties of carbon, PANi and metal nanostructures, many excellent electrical and electrochemical properties are obtained.^10,24,25^ Moreover, new methods for the synthesis of nanostructured metals embedded in nanocarbon and nano-PANi structures will open interesting opportunities in advanced applications based on carbon/PANi/metal nanocomposites like electrochemical DNA sensors.^24,25^ Electrochemical DNA sensors work based on changes in electrochemical signals caused by interactions between probe and target DNA strands on the electrode surface. The successful development of an electrochemical DNA sensor depends on the design and fabrication of electrodes, on the electrode surface modification with an intermediate material layer to improve the efficiency of the DNA probe immobilization on the electrode surface, and on the construction of electrochemical measurement processes to detect DNA target strands in analytical samples. In this work, a three-step electrochemical procedure was conducted to synthesize directly a sandwich-structured electrochemically reduced graphene oxide/polyaniline nanowires/silver nanoflowers (ERGO/PANi NWs/AgNFs) nanocomposite on a Pt microelectrode. Then the optimized ERGO/PANi NWs/AgNFs nanocomposite was used for the first time to develop an electrochemical DNA sensor. The combination among ERGO, PANi NWs and AgNFs was expected to facilitate the DNA probe immobilization and improve the electrochemical signals of the DNA sensor. Electrochemical impedance spectroscopy (EIS) technique was performed using the fabricated DNA sensor to detect DNA target strands with low detection limit and high selectivity.

## 2. Experiment

### 2.1. Chemicals and instrumentations

#### 2.1.1. Chemicals

Graphite powder, aniline (C_6_H_5_NH_2_, 99.5 wt%), silver nitrate (AgNO_3_, 99 wt%), phosphate buffer solution (PBS), potassium hexacyanoferrate(iii) (K_3_Fe(CN)_6_, 99 wt%), potassium hexacyanoferrate(ii) trihydrate (K_4_Fe(CN)_6_·3H_2_O, 99.95 wt%) and potassium nitrate (KNO_3_, 99 wt%) were purchased from Sigma Aldrich. The supporting chemicals, including sodium nitrate (NaNO_3_, 99 wt%), hydrogen peroxide (H_2_O_2_, 30 wt%), hydrochloric acid (HCl, 37 wt%), potassium permanganate (KMnO_4_, 99 wt%), nitrogen (N_2_, 99.9 wt%), potassium dichromate (K_2_Cr_2_O_7_, 99 wt%) and sulfuric acid (H_2_SO_4_, 98 wt%) were of analytical grade. The DNA probe and the complementary and non-complementary DNA target strands were purchased from Integrated DNA Technologies (IDT). These single-stranded DNA sequences are listed in Table 1.

**Table 1:** The DNA probe and the complementary and non-complementary DNA target strands

Probe	3′-CTGCATGGTACCTCTGACCTCCAGA-5′
Complementary target	3′-TCTGGAGGTCAGAGGTACCATGCAG-5′
Non-complementary target	3′-ACTGAGAACGTGGGCATGAGTCGCA-5′

#### 2.1.2. Integrated Pt microelectrodes

The integrated Pt microelectrode consisting of a 0.80 mm^2^ working electrode (WE) and a 5.00 mm^2^ counter electrode (CE), was deposited on a SiO_2_/Si substrate using the cathode sputtering technique with the configuration and fabrication process discussed in our previous work.^26^

#### 2.1.3. Instrumentations

Electrosynthesis of the ERGO/PANi NWs/AgNFs nanocomposite and electrochemical measurements were performed using a PGSTAT302N AutoLab electrochemical workstation (Metrohm, Netherlands) and a three-electrode configuration consisting of the WE and the CE which were integrated in the fabricated Pt microelectrode and an Ag/AgCl electrode (SCE) in 3 M KCl solution as a reference electrode (RE). Scanning electron microscopy (SEM) images and energy dispersive X-ray spectroscopy (EDX) spectra of GO, ERGO, ERGO/PANi NWs and ERGO/PANi NWs/AgNFs were recorded with a Nova NanoSEM 450 microscope (FEI Company, Netherlands). Fourier transform infrared spectroscopy (FT-IR) spectra of GO and ERGO were investigated using an IRAffinity-1S FTIR spectrometer (Shimadzu, Japan). Raman spectra of GO, ERGO, PANi NWs and ERGO/PANi NWs were measured using a LabRAM HR 800 Raman (Horiba Jobin Yvon, France).

### 2.2. Electrosynthesis of ERGO on Pt electrodes

GO was synthesized in our laboratory by Hummer's method as previously reported.^27^ Before the preparation of ERGO on the Pt electrodes, these electrodes (as the working electrodes) were cleaned in a saturated solution of K_2_Cr_2_O_7_/H_2_SO_4_, and were electrochemically activated in 0.5 M H_2_SO_4_ solution using the cyclic voltammetry (CV) method with the scan rate of 100 mV s^−1^ and the voltage range of 0–1.5 V until the CV characteristics were stable. Then 5 μL of a 0.5 mg mL^−1^ GO suspension was drop-casted on each of the Pt microelectrodes. After that, the ERGO layer was fabricated directly on the working electrodes using the CV method. This step was conducted in PBS (pH 7.4) at the voltage range from 0.1 to −1.2 V *vs.* Ag/AgCl RE for 8 cycles and the scan rate of 50 mV s^−1^.

### 2.3. Electrosynthesis of PANi NWs on Pt/ERGO electrodes

An electrolyte solution consisting of 0.05 M aniline monomer and 0.5 M H_2_SO_4_ was blown with N_2_ gas for 10 minutes to remove the dissolved oxygen. PANi NWs were electropolymerized on the Pt/ERGO electrodes using the chronoamperometry (CA) method with an applied voltage of 0.9 V *vs.* Ag/AgCl RE. Then the Pt/ERGO/PANi NWs electrodes were washed with deionized water and were dried at room temperature (RT).

### 2.4. Electrosynthesis of AgNFs on Pt/ERGO/PANi NWs electrodes

For electrosynthesis of AgNFs onto the Pt/ERGO/PANi NWs electrodes, an electrolyte solution consisting of 0.001 M AgNO_3_ and 0.1 M KNO_3_ was used, and the CA method was performed with an applied voltage of −1.2 V *vs.* Ag/AgCl RE. After that, deionized water was used to clean the Pt/ERGO/PANi NWs/AgNFs electrodes. Then these electrodes were dried at RT.

### 2.5. Immobilization of DNA probe on Pt/ERGO/PANi NWs/AgNFs electrodes

For DNA probe immobilization, 5 μL of a 50 μM DNA probe solution in PBS (pH 7.4) was dropped onto each of the Pt/ERGO/PANi NWs/AgNFs electrodes. The DNA probe immobilization was kept for 1 hour at RT. The electrodes were then cleaned with deionized water to remove DNA probe strands which had weak linkages with the ERGO/PANi NWs/AgNFs material. After that, the DNA sensors were dried by a N_2_ gas stream and were ready for further measurements.

### 2.6. Detection of DNA target using ERGO/PANi NWs/AgNFs-based electrochemical biosensors

For DNA target detection, each of the DNA sensors was dropped with 5 μL of complementary DNA target solutions with different concentrations (from 1.0 × 10^−14^ M to 1.0 × 10^−7^ M) in PBS (pH 7.4). The DNA hybridization was conducted for 1 hour at RT. After that, electrochemical impedance spectroscopy (EIS) spectra of the DNA sensors were recorded in a solution consisting of K_3_Fe(CN)_6_/K_4_Fe(CN)_6_ (0.005 M) and 0.1 M KNO_3_ in PBS (pH 7.4) at a frequency range from 10^5^ Hz to 0.1 Hz, a DC potential of 160 mV and an AC potential of 5 mV (*vs.* Ag/AgCl RE). On the EIS spectra, the increase in the electron transfer resistance Δ*R*_ct_ (Δ*R*_ct_ = *R*_ct,i_ − *R*_ct,0_), where *R*_ct,i_ and *R*_ct,0_ are the electron transfer resistances in the presence and absence of the DNA target, respectively, is used as the DNA hybridization signal.

## 3. Results and discussion

### 3.1. Characterization of ERGO electrosynthesized on Pt electrodes

**Fig. 1:**
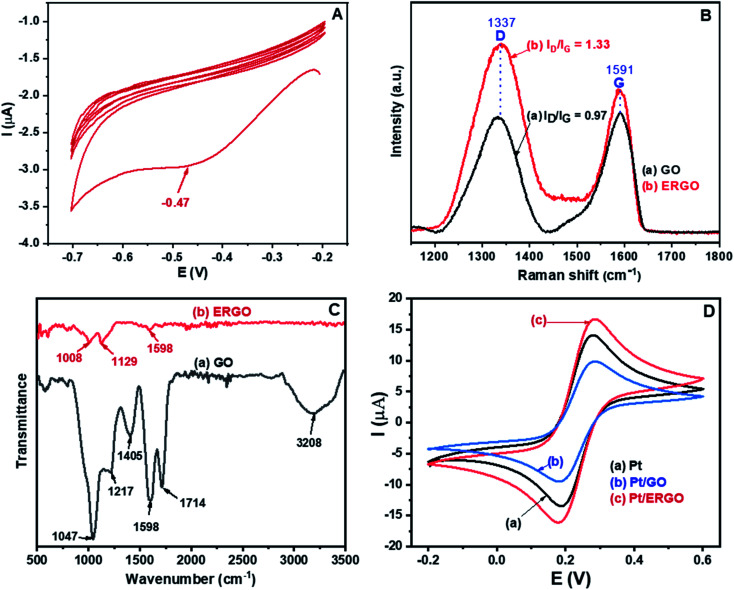
(A) CV result corresponding to the reduction of GO drop-casted on the Pt electrode; (B) Raman spectra and (C) FT-IR spectra of: (a) GO and (b) ERGO electrosynthesized on Pt electrodes; (D) CV results of: (a) Pt, (b) Pt/GO, and (c) Pt/ERGO electrodes recorded in K_3_Fe(CN)_6_/K_4_Fe(CN)_6_ (0.005 M) and 0.1 M KNO_3_ solution at 25 mV s^−1^ scan rate.

Fig. 1A shows the CV result corresponding to the reduction of the drop-casted graphene oxide (GO) on the Pt microelectrode. As can be seen in Fig. 1A, in the first cycle, a reduction peak appears at −0.47 V. Thus, the GO material was electrochemically reduced, and an ERGO layer was formed directly on the Pt electrode. However, as the amount of GO was very small, only 5 μL of the 0.5 mg mL^−1^ GO suspension drop-casted on the Pt microelectrode, the intensity of the reduction peak decreases significantly and this reduction peak almost disappears in the subsequent cycles. Besides, for all cycles, there are no oxidation peaks. This result indicates that the electrochemical transition from GO to its reduced form was irreversible and GO was effectively converted into ERGO on the Pt electrode.^28^

The Raman results of the fabricated GO and ERGO materials are shown in Fig. 1B. The peaks at 1337 cm^−1^ and 1591 cm^−1^ are attributed to the typical D and G bands of GO and ERGO, respectively.^29^ The D band is associated with defects and disorders of carbon, and the G band is assigned to E_2g_ mode of sp^2^ hybridized carbon relating to the degree of graphitization.^30,31^ The increase in the intensity ratio *I*_D_/*I*_G_ exhibits the increase in the number of defects and disorders caused by the removal of functional groups from GO. Therefore, this is an effective signal of the transition from GO to ERGO.^29,32^ It is clearly seen that in the Raman spectrum of ERGO, Fig. 1B (curve b), the value of *I*_D_/*I*_G_ (1.33) is significantly higher than that in the Raman spectrum of GO (0.97), Fig. 1B (curve a). Thus, GO was electrochemically reduced to ERGO on the Pt microelectrodes.

Fig. 1C shows the FT-IR spectra of the synthesized GO and ERGO materials. The FT-IR spectrum of GO, Fig. 1C (curve a), exhibits the bands at 3208 and 1714 cm^−1^, which are assigned to the stretching vibrations of O–H and C=O (in COOH), respectively.^32^ The bands at 1047, 1217 and 1405 cm^−1^ are associated with the C–O, C–O–C and O–H (C–OH) stretching vibrations, respectively.^32–34^ Besides, the band at 1598 cm^−1^ is attributed to the C=C stretching mode.^33^ On the other hand, in the FT-IR spectrum of ERGO, Fig. 1C (curve b), the bands at 3208, 1714, 1047, 1217 and 1405 cm^−1^ disappear. The ERGO spectrum reveals only three bands at 1008, 1129 and 1598 cm^−1^, corresponding to the C–O and C=C stretching vibrations.^34,35^ These results demonstrate that GO was effectively reduced to form ERGO.

The cyclic voltammograms measured in K_3_Fe(CN)_6_/K_4_Fe(CN)_6_ (0.005 M) and 0.1 M KNO_3_ solution at 25 mV s^−1^ scan rate of (a) Pt, (b) Pt/GO, and (c) Pt/ERGO electrodes are shown in Fig. 1D. For the three different CV results, no changes in the potentials related to the oxidation of Fe(CN)_6_^4−^ and the reduction of Fe(CN)_6_^3−^ are observed, demonstrating that the electrochemical behavior of ERGO formed on the WE is stable. On the other hand, as can be seen in Fig. 1D, the peak current of GO, Fig. 1D (curve b), is even lower than that of the bare Pt electrode, Fig. 1D (curve a), due to GO's high resistivity caused by the oxygen-containing functional groups. Meanwhile, ERGO loses these groups, it exhibits good conductivity and does not change the electron transfer kinetics of the Fe(CN)_6_^3−/4−^ redox couple.^36^ Therefore, the peak current corresponding to the Pt/ERGO case, Fig. 1D (curve c), is higher than those in the Pt and Pt/GO cases. This result indicates that ERGO has higher electrochemical activity than that of GO and the Pt microelectrode was effectively modified with ERGO.

**Fig. 2:**
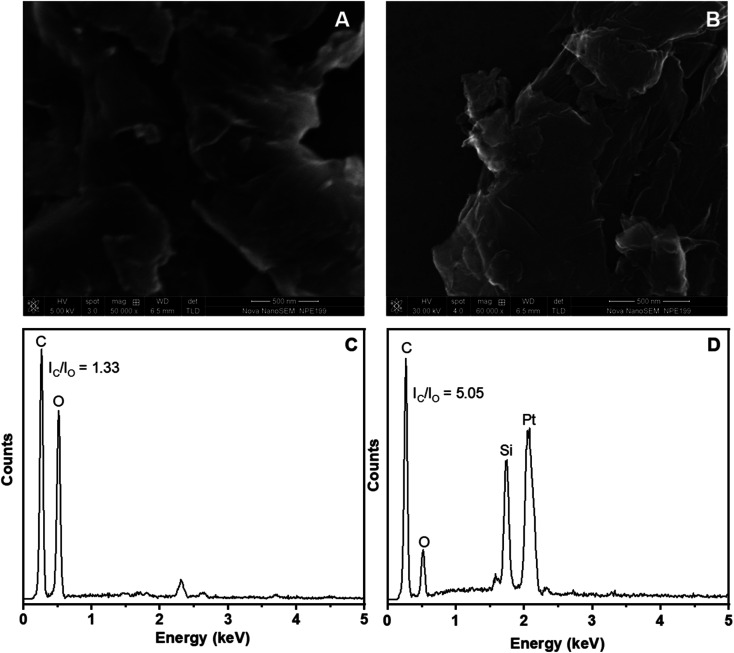
(A) and (C): SEM image and EDX spectrum of GO; (B) and (D): SEM image and EDX spectrum of ERGO electrosynthesized on Pt electrodes.

Fig. 2 shows the SEM images and the EDX spectra of the fabricated GO and ERGO materials. As can be seen in the SEM images of GO and ERGO, Fig. 2A and B, these materials contain thin and crumpled sheets which are randomly aggregated and closely associated with each other. Besides, in the EDX spectra of GO and ERGO, Fig. 2C and D, carbon and oxygen elements are observed at 0.27 and 0.54 keV, respectively. In the EDX spectrum of ERGO, Fig. 2D, the intensity ratio *I*_C_/*I*_O_ (5.05) increases significantly compared to that in the EDX spectrum of GO (1.33), Fig. 2C. These results further confirm that ERGO was successfully synthesized from GO which was drop-casted on the Pt microelectrode.

### 3.2. Characterization of PANi NWs electrosynthesized on Pt/ERGO electrodes

**Fig. 3:**
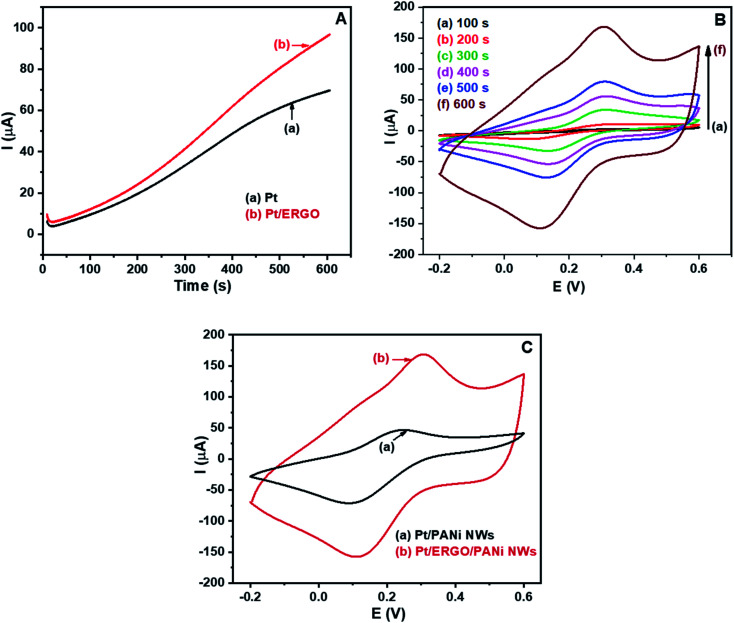
(A) CA results of: (a) Pt, and (b) Pt/ERGO electrodes performed in 0.05 M aniline and 0.5 M H_2_SO_4_ solution; (B) CV results of Pt/ERGO/PANi NWs electrodes in which PANi NWs were electropolymerized using CA method with different times: (a) 100, (b) 200, (c) 300, (d) 400, (e) 500, and (f) 600 seconds; (C) CV results of: (a) Pt/PANi NWs, and (b) Pt/ERGO/PANi NWs electrodes. Experimental conditions: CV measurements were conducted in K_3_Fe(CN)_6_/K_4_Fe(CN)_6_ (0.005 M) and 0.1 M KNO_3_ solution at 25 mV s^−1^ scan rate.

After the electrosynthesis of ERGO on the Pt electrode, PANi NWs were electrosynthesized directly on the Pt/ERGO electrode using the CA method. Fig. 3A shows the CA results of (a) Pt and (b) Pt/ERGO electrodes performed in the electrolyte solution consisting of 0.05 M aniline and 0.5 M H_2_SO_4_. As can be seen in Fig. 3A, for the case of the Pt/ERGO electrode, Fig. 3A (curve b), the current intensity is higher than that of the Pt electrode, Fig. 3A (curve a). This result is obtained due to the high electrochemical activity of ERGO, hence the electrosynthesis of PANi NWs on the Pt/ERGO electrode is more effective than that on the Pt electrode.

The electropolymerization processes of PANi NWs on the Pt/ERGO electrodes were conducted in 100, 200, 300, 400, 500 and 600 seconds. Cyclic voltammograms of the fabricated Pt/ERGO/PANi NWs electrodes in K_3_Fe(CN)_6_/K_4_Fe(CN)_6_ (0.005 M) and 0.1 M KNO_3_ solution at 25 mV s^−1^ scan rate are shown in Fig. 3B. As can be seen in Fig. 3B (curve a to f), when the polymerization time of PANi NWs increases from 100 to 600 seconds, the currents of anodic peaks increase whereas the currents of cathodic peaks decrease. When the electrolysis time is 600 seconds, Fig. 3B (curve f), the highest current density is observed. The high conductivity of the ERGO/PANi NWs material would facilitate the data treatment, therefore, for further electrodeposition processes, the selected time was 600 seconds.

In Fig. 3C, the CV results of the Pt/PANi NWs and Pt/ERGO/PANi NWs electrodes performed in K_3_Fe(CN)_6_/K_4_Fe(CN)_6_ (0.005 M) and 0.1 M KNO_3_ solution at 25 mV s^−1^ scan rate are shown. In case of the Pt/ERGO/PANi NWs electrode, Fig. 3C (curve b), the peak current is higher than that of the Pt/PANi NWs electrode, Fig. 3C (curve a). This result is due to the larger surface area and the higher conductivity of the ERGO/PANi NWs material compared to the individual PANi NWs.

**Fig. 4:**
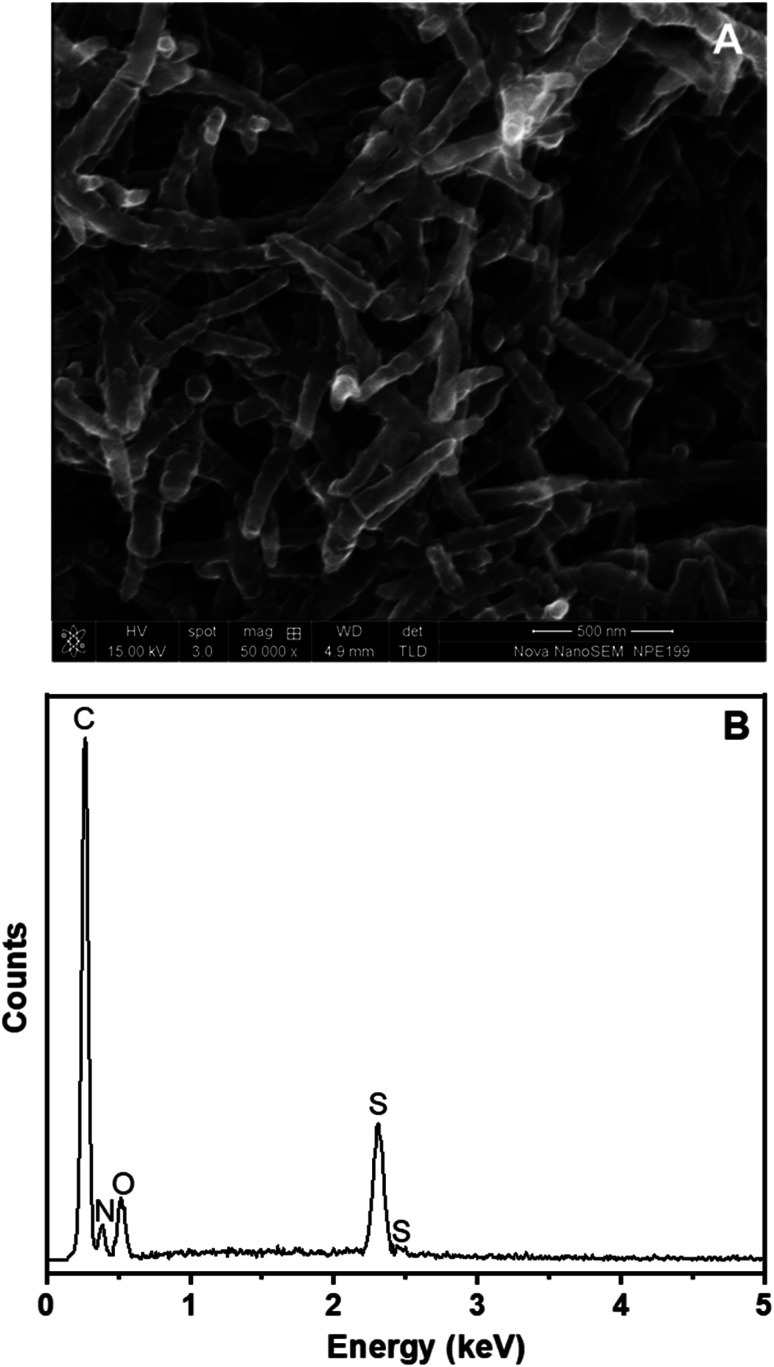
(A) SEM image and (B) EDX spectrum of PANi NWs electropolymerized on Pt/ERGO electrodes.

The SEM image of PANi NWs formed directly on the surface of the Pt/ERGO electrode is exhibited in Fig. 4A. PANi NWs with 55–80 nm diameters are distributed throughout the surface of the working electrode. The size of PANi NWs is uniform, and the nanowires are smooth, homogenous and less agglomerated.

The EDX spectrum in Fig. 4B shows relatively the composition of PANi NWs electrosynthesized on the Pt/ERGO electrode. Carbon element, which is a constituent of both PANi and ERGO materials, is observed at 0.27 keV. Nitrogen element, a constituent of PANi NWs, appears at 0.39 keV. Moreover, the appearance of sulfur element at 2.31 keV indicates that H_2_SO_4_ was doped into PANi. Doping PANi with H_2_SO_4_ can improve the conductivity of PANi which is due to the cations (the charge carriers) formed at the imine nitrogen atoms.^37,38^ Besides, oxygen element, a constituent of both PANi NWs doped with H_2_SO_4_ and ERGO, is also observed at 0.54 keV.

**Fig. 5:**
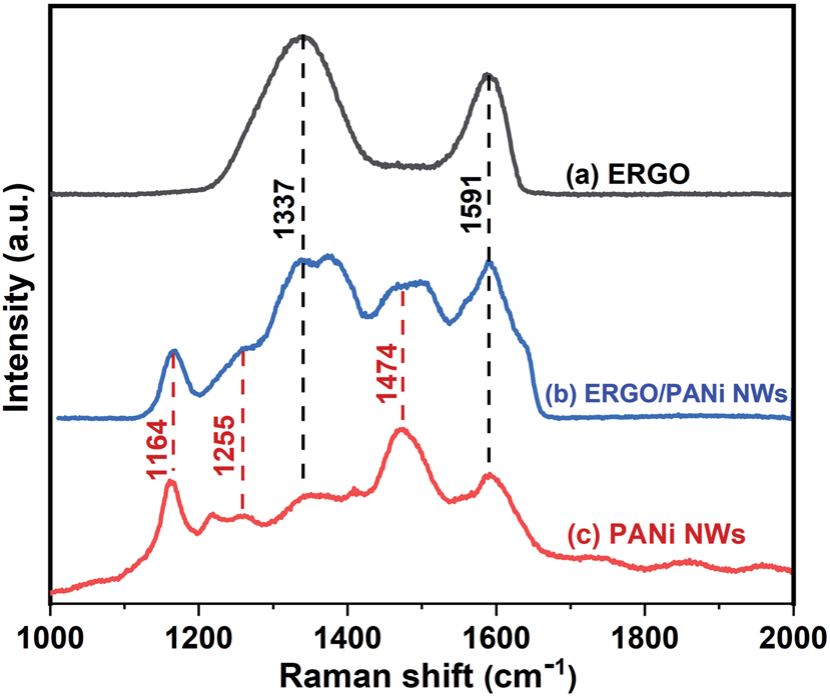
Raman spectra of: (a) ERGO, (b) ERGO/PANi NWs, and (c) PANi NWs electrosynthesized on Pt electrodes.

Fig. 5 exhibits the Raman spectra of ERGO, PANi NWs and ERGO/PANi NWs electrosynthesized on the Pt electrodes. The Raman characteristic peaks of both ERGO, Fig. 5 (curve a), and PANi NWs, Fig. 5 (curve c), are observed in Fig. 5 (curve b) which is the Raman spectrum of ERGO/PANi NWs. As shown in Fig. 5 (curve c and curve b), the bands at 1164 and 1591 cm^−1^ are assigned to the C–H bending vibration of the quinoid/benzenoid rings and the C–C stretching of the benzenoid rings, respectively.^39^ The bands at 1255, 1337 and 1474 cm^−1^ are attributed to the C–N stretching mode of polaronic units, the C–N^+^ vibration of polaronic structures and the C=N stretching of the quinoid rings, respectively.^39,40^ Besides, as can be seen in Fig. 5 (curve b), the peaks at 1337 and 1591 cm^−1^ are in the overlap regions between ERGO, Fig. 5 (curve a), and PANi NWs, Fig. 5 (curve c). As a result, in Fig. 5 (curve b), the intensities of these peaks are higher than those in Fig. 5 (curve c). These results demonstrate that the ERGO/PANi NWs material was successfully electrochemical-synthesized on the Pt microelectrodes.

### 3.3. Characterization of AgNFs electrosynthesized on Pt/ERGO/PANi NWs electrodes

**Fig. 6:**
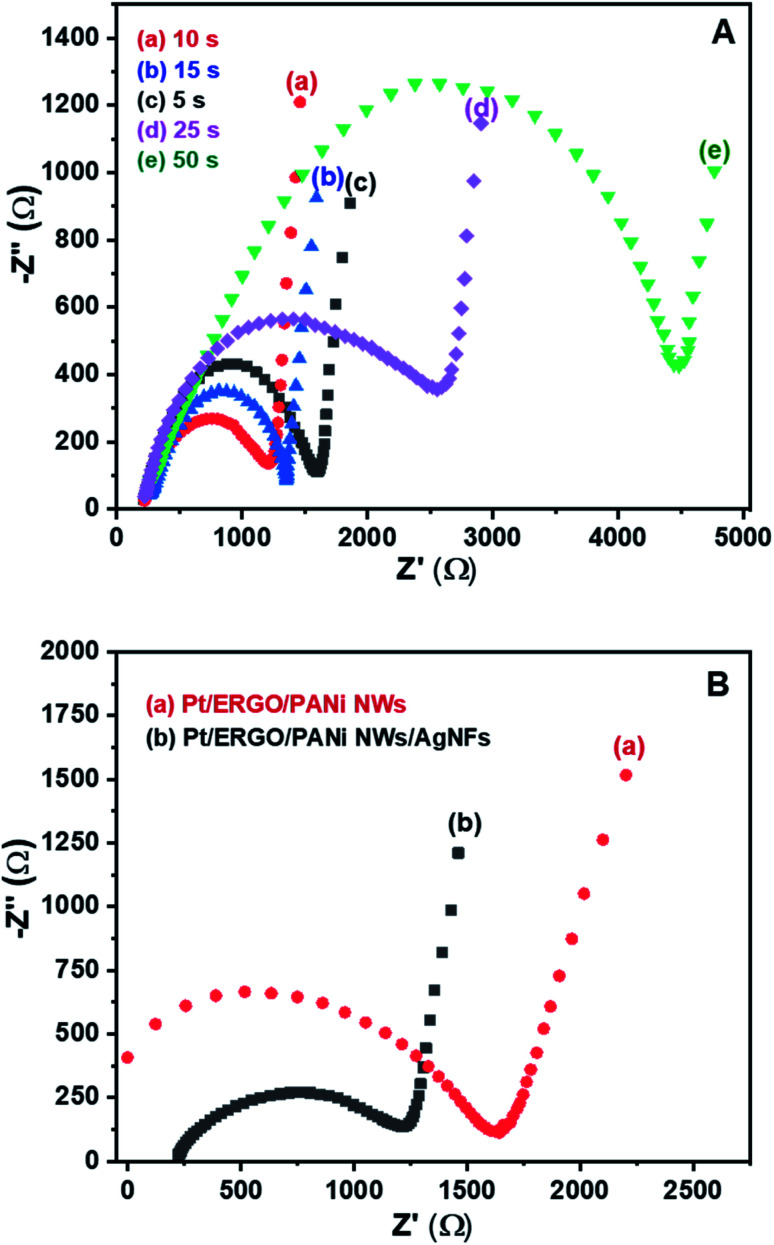
(A) EIS spectra (Nyquist plots) of Pt/ERGO/PANi NWs/AgNFs electrodes in which AgNFs were electrodeposited using CA method with different times: (a) 10, (b) 15, (c) 5, (d) 25, and (e) 50 seconds; (B) EIS spectra (Nyquist plots) of (a) Pt/ERGO/PANi NWs, and (b) Pt/ERGO/PANi NWs/AgNFs electrodes. Experimental conditions: EIS spectra were measured in K_3_Fe(CN)_6_/K_4_Fe(CN)_6_ (0.005 M) and 0.1 M KNO_3_ solution, frequency range: 100 kHz to 100 mHz, *E*_AC_ = 5 mV, *E*_DC_ = 160 mV.

AgNFs were electrosynthesized directly onto the Pt/ERGO/PANi NWs electrodes using the CA method. Fig. 6A shows the EIS spectra in Nyquist form of the Pt/ERGO/PANi NWs/AgNFs electrodes measured in K_3_Fe(CN)_6_/K_4_Fe(CN)_6_ (0.005 M) and 0.1 M KNO_3_ solution, corresponding to the different times of the CA processes: 5, 10, 15, 25 and 50 seconds. As can be seen in Fig. 6A, the EIS spectra of the Pt/ERGO/PANi NWs/AgNFs electrodes consist of a semicircle characterizing a charge transfer process and a linear region characterizing a diffusion process. In which, the charge transfer resistance (*R*_ct_) relating to changes of the interface of the electrode can be determined by the diameter of the semicircle.^33^

In Fig. 6A, the *R*_ct_ value is the smallest when the CA time is 10 seconds, Fig. 6A (curve a). When the CA time is less than 10 seconds, Fig. 6A (curve c), a large number of Ag^+^ ions remain existing in the electrolyte solution, so the amount of AgNFs formed on the Pt/ERGO/PANi NWs electrode is limited. When the CA time increases from 5 to 10 seconds, the amount of AgNFs increases, leading to the decrease in the *R*_ct_ value of the Pt/ERGO/PANi NWs/AgNFs electrode. This is because the high conductivity of the sandwich arrangement of AgNFs on ERGO/PANi NWs facilitates the electron transfer. However, when the CA time increases from 10 to 50 seconds, Fig. 6A (curve b, d, and e), the *R*_ct_ increases and reaches its maximum value at 50 seconds. Thus, the further increase in the CA time leads to the agglomeration of AgNFs (the formation of a silver film) and to the increase in the *R*_ct_ of the Fe(CN)_6_^3−/4−^ redox couple to the Pt electrode. Therefore, for the further electrosynthesis of AgNFs on the Pt/ERGO/PANi NWs electrodes, the optimized CA time was 10 seconds.

Fig. 6B shows the EIS spectra measured in K_3_Fe(CN)_6_/K_4_Fe(CN)_6_ (0.005 M) and 0.1 M KNO_3_ solution of the Pt/ERGO/PANi NWs and Pt/ERGO/PANi NWs/AgNFs electrodes. It can be seen that, the *R*_ct_ value of the Pt/ERGO/PANi NWs/AgNFs electrode (975 Ω, Fig. 6B (curve b)) is lower than that of the Pt/ERGO/PANi NWs electrode (1618 Ω, Fig. 6B (curve a)). This result indicates that AgNFs were successfully synthesized on the Pt/ERGO/PANi NWs electrode surface. The ERGO/PANi NWs material plays an important role as a skeleton supporting to the formation of AgNFs that have high electrochemical activity. Moreover, when the electrosynthesis method was applied, the good adhesion between AgNFs and ERGO/PANi NWs can be obtained, so the good ohmic contact between the material layers was ensured, and the *R*_ct_ value of the Pt/ERGO/PANi NWs/AgNFs electrode reduced.

**Fig. 7:**
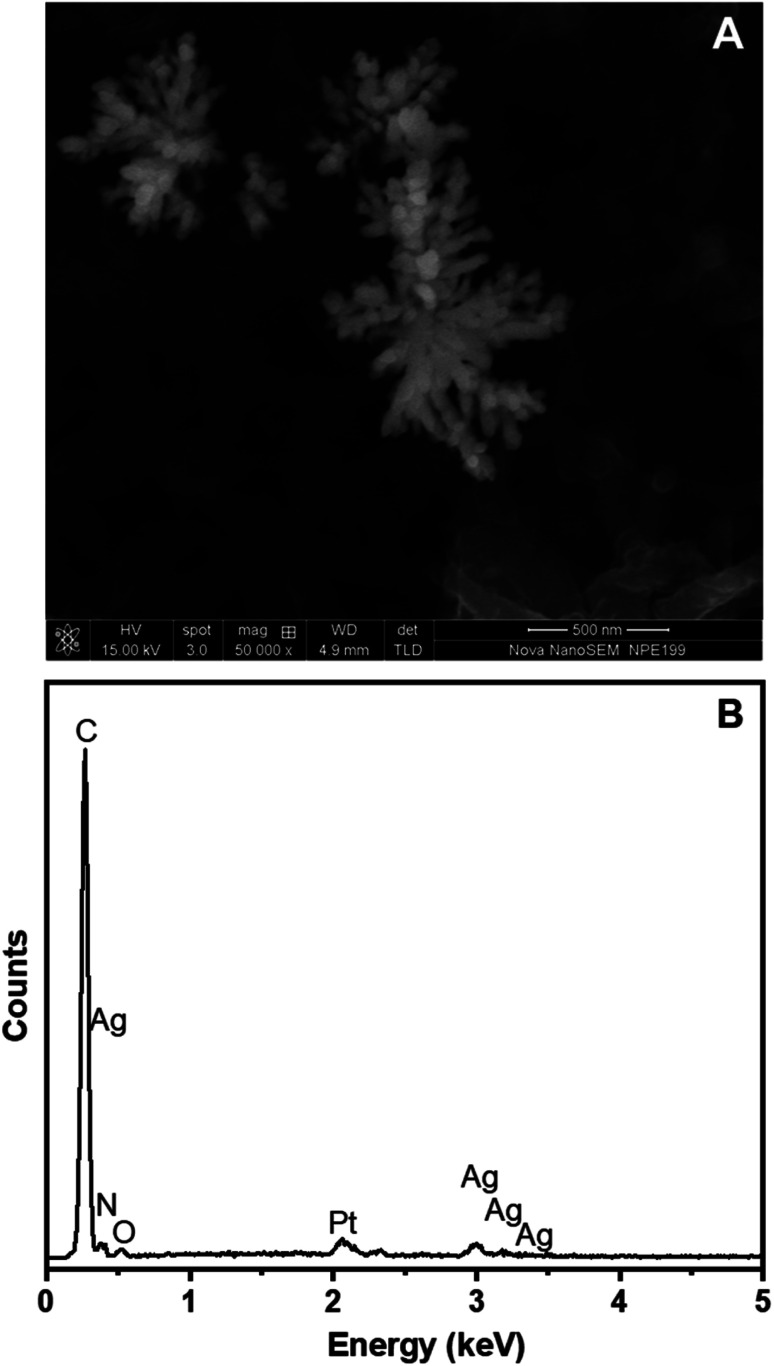
(A) SEM image and (B) EDX spectrum of AgNFs electrodeposited on Pt/ERGO/PANi NWs electrodes.

Fig. 7A shows the SEM image of AgNFs formed on the surface of the Pt/ERGO/PANi NWs electrode, in which AgNFs were electrosynthesized using the CA method with the electrodeposition time of 10 seconds. It is clearly seen that Ag nanoflowers contain nanorods which are originated from one point, and arranged freely outwards. These nanorods look like petals on a flower, and their diameters range from 20 to 30 nm. The electrodeposition method allows direct fabrication of AgNFs onto the ERGO/PANi NWs surface, and the ERGO/PANi NWs layer has an important role as a substrate facilitating the formation of AgNFs.

The EDX spectrum of ERGO/PANi NWs/AgNFs electrosynthesized on the Pt electrodes is illustrated in Fig. 7B. As can be seen in Fig. 7B, carbon, nitrogen, oxygen, and sulfur elements, which characterize for the ERGO/PANi NWs material, are observed at 0.27, 0.39, 0.54 and 2.31 keV, respectively. These peaks are similar to the results shown in Fig. 4B. Particularly, in Fig. 7B, the peaks which are associated with silver do appear at 0.29, 2.99, 3.18 and 3.38 keV. Thus, the SEM and EDX results further confirm that AgNFs were electrosynthesized on the Pt/ERGO/PANi NWs electrodes.

In summary, the sandwich-structured ERGO/PANi NWs/AgNFs nanocomposite was successfully synthesized on the Pt microelectrode using the three-step electrochemical procedure and the effects of each component on the electrochemical properties of the material were also evaluated. Firstly, the Pt microelectrode was effectively modified with ERGO. Then, thanks to the high electrochemical activity of ERGO (Fig. 1D), the electrosynthesis of PANi NWs on the Pt/ERGO electrode was more effective compared to that on the Pt electrode (Fig. 3A and C). The obtained PANi NWs with large surface area and high conductivity were expected to facilitate the DNA probe immobilization because phosphate groups of DNA probe strands can form linkages with amino groups of PANi NWs.^41^ Finally, the ERGO/PANi NWs material acted as a skeleton supporting to the formation of AgNFs that have high electrochemical activity (Fig. 6B), so the electrochemical signals of the DNA sensor were expected to be improved.

### 3.4. Direct immobilization of DNA probe on Pt/ERGO/PANi NWs/AgNFs electrodes

**Fig. 8:**
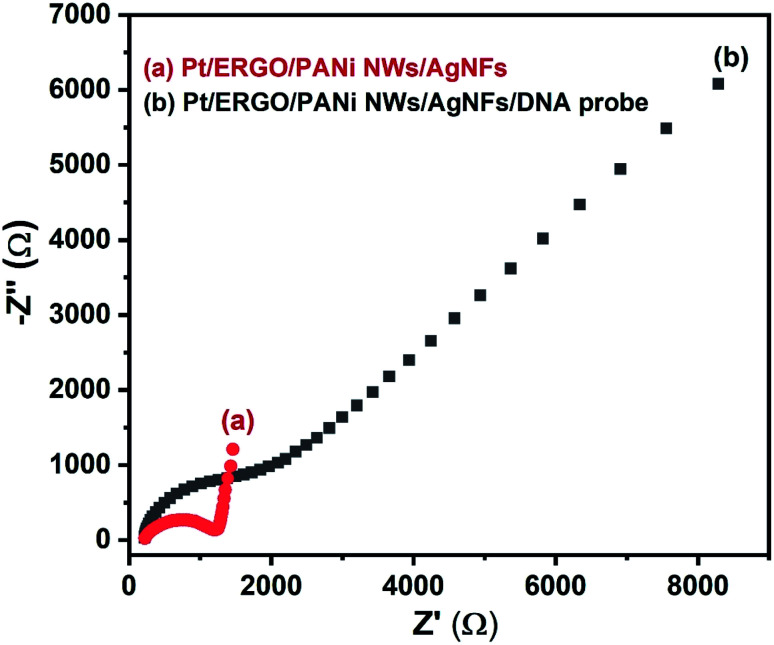
EIS spectra (Nyquist plots) of (a) Pt/ERGO/PANi NWs/AgNFs, and (b) Pt/ERGO/PANi NWs/AgNFs/DNA probe electrodes. Experimental conditions: EIS spectra were measured in PBS buffer (pH 7.4) solution consisting K_3_Fe(CN)_6_/K_4_Fe(CN)_6_ (0.005 M) and 0.1 M KNO_3_, frequency range: 100 kHz to 100 mHz, *E*_AC_ = 5 mV and *E*_DC_ = 160 mV.

The EIS spectra of the Pt/ERGO/PANi NWs/AgNFs and Pt/ERGO/PANi NWs/AgNFs/DNA probe electrodes are shown in Fig. 8. The impedance of the Pt/ERGO/PANi NWs/AgNFs/DNA probe electrode, Fig. 8 (curve b), increases significantly compared to that in the case of the Pt/ERGO/PANi NWs/AgNFs electrode, Fig. 8 (curve a). The direct immobilization of DNA probe on the Pt/ERGO/PANi NWs/AgNFs electrode was performed effectively, leading to the increase in the *R*_ct_ value, from 975 Ω (Fig. 8, curve a) to 2390 Ω (Fig. 8, curve b). The PANi nanowire structure with porous surface characteristics, homogenous distribution, large surface area and high conductivity plays an important role in the immobilization of DNA probe strands on the electrode surface thanks to the formation of linkages between phosphate groups of DNA probe strands and amino groups of PANi NWs.^41^ Therefore, in this study, the DNA probe immobilization conducted with PANi NWs is more simple, rapid and effective in comparison with other complex immobilization processes using PANi films.^8,42^

### 3.5. DNA hybridization detection using ERGO/PANi NWs/AgNFs-based electrochemical DNA sensors

**Fig. 9:**
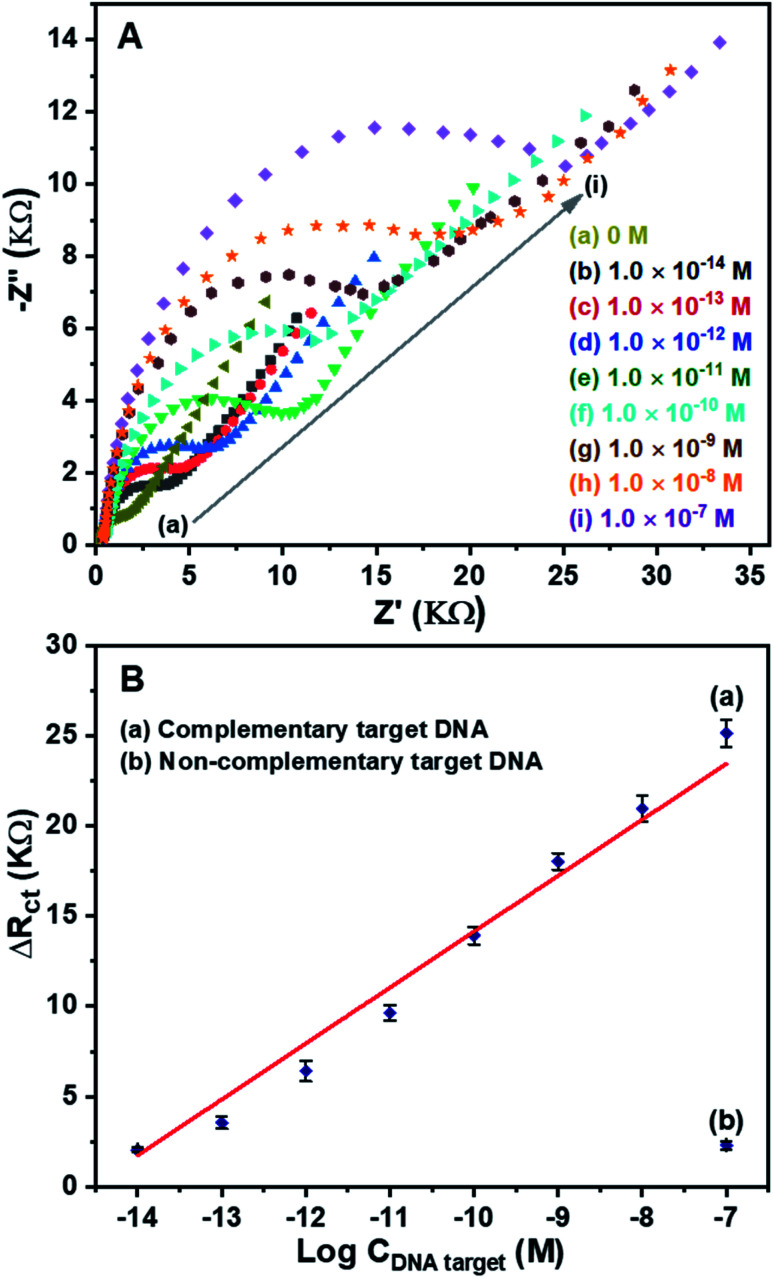
(A) EIS spectra (Nyquist plots) of the DNA sensors (the Pt/ERGO/PANi NWs/AgNFs/DNA probe electrodes) after hybridization with the complementary target DNA corresponding to different concentrations: (a) 0 M, (b) 1.0 × 10^−14^ M, (c) 1.0 × 10^−13^ M, (d) 1.0 × 10^−12^ M, (e) 1.0 × 10^−11^ M, (f) 1.0 × 10^−10^ M, (g) 1.0 × 10^−9^ M, (h) 1.0 × 10^−8^ M, and (i) 1.0 × 10^−7^ M. Experimental conditions: EIS spectra were measured in PBS buffer (pH 7.4) solution consisting K_3_Fe(CN)_6_/K_4_Fe(CN)_6_ (0.005 M) and 0.1 M KNO_3_; (B) Response of the DNA sensors to: (a) different concentrations of complementary target DNA, and (b) 1.0 × 10^−7^ M of non-complementary target DNA.

For detection of DNA target, EIS measurements were conducted due to their sensitivity to impedance changes caused by DNA hybridization happening at the surface–electrolyte interface.^33^ The EIS spectra of the DNA sensors after hybridization with the complementary target DNA at different concentrations are shown in Fig. 9A. In the absence of the DNA target, Fig. 9A (curve a), the lowest impedance is obtained. On the other hand, in the presence of the DNA target, Fig. 9A (curve b to i), the higher impedances are observed. The increase in the concentration of the DNA target leads to the increase in the impedance of the DNA sensors. The results demonstrate that the DNA hybridization happened at the surface of the ERGO/PANi NWs/Ag NFs material, so the charge transfer resistance increased, leading to the increase in the impedance. Thus, the fabricated DNA sensors can be used to effectively detect the complementary target DNA.

For investigation of the sensitivity of the DNA sensors, the Δ*R*_ct_ values at different concentrations of the complementary target DNA were calculated. Fig. 9B (curve a) shows a good linear relationship between the Δ*R*_ct_ and the logarithm of the complementary target DNA concentration in the range from 1.0 × 10^−14^ M to 1.0 × 10^−7^ M. The obtained linear equation is Δ*R*_ct_ (KΩ) = 3.0999 log *C* + 45.1436 with the correlation coefficient of *R*^2^ = 0.9846. The detection limit is 2.70 × 10^−15^ M, using the LOD calculation.^43^ For investigation of the selectivity of the DNA sensors, the Δ*R*_ct_ value corresponding to 1.0 × 10^−7^ M of the noncomplementary target DNA was calculated, Fig. 9B (curve b). As can be seen in Fig. 9B (curve b), the response signal is negligible at the highest studied concentration (1.0 × 10^−7^ M) of the noncomplementary target DNA. The results reveal that the developed DNA sensors using ERGO/PANi NWs/AgNFs layers have high sensitivity and good selectivity.

The comparison of this fabricated DNA sensor with some others in the previous studies is shown in Table 2. Particularly, our previous study and some studies in the literature on electrochemical DNA sensors based on materials such as PANi NWs/AgNPs,^15^ ERGO,^44^ PANi NWs,^41^ GO/AgNPs,^45^ and ERGO/PANi^46^ are also listed in Table 2 to compare their DNA detection efficiency with that of the ERGO/PANi NWs/AgNFs-based sensor in this work. The electrochemical DNA sensor based on the ERGO/PANi NWs/AgNFs nanocomposite exhibited promising results, such as simple immobilization of DNA probe on the electrode surface, direct detection, and high sensitivity (with the low detection limit, 2.70 × 10^−15^ M). Moreover, the DNA sensor based on the Pt/ERGO/PANi NWs/AgNFs microelectrodes required very small volumes of both the DNA probe and DNA target samples (only 5 μL) for the DNA probe immobilization and the DNA target detection, respectively. In addition, the Pt/ERGO/PANi NWs/AgNFs microelectrodes and the electrochemical methods will also facilitate the fabrication of lab-on-a-chip devices.^9,47^

**Table 2:** Comparison of this fabricated DNA sensor with some others in the literature

Surface modification	Synthesis method	Linear range (M)	Detection limit (M)	Reference
ERGO	Drop-casting;	1.0 × 10^−12^–1.0 × 10^−9^	3.0 × 10^−13^	44
	Electrochemical method			
PANi NWs	Three-step electrochemical method	2.25 × 10^−12^–2.25 × 10^−10^	1.0 × 10^−12^	41
ERGO/PANi	Drop-casting;	1.0 × 10^−13^–1.0 × 10^−7^	3.2 × 10^−14^	46
	Electrochemical method			
GO/AgNPs	Chemical method	1.0 × 10^−14^–1.0 × 10^−8^	7.6 × 10^−15^	45
PANi NWs/AgNPs	Two-step electrochemical method	1.0 × 10^−14^–1.0 × 10^−9^	2.80 × 10^−15^	15
PANi/Graphene sheets	Electrochemical method;	1.0 × 10^−13^–1.0 × 10^−6^	1.0 × 10^−14^	48
	Drop-casting			
Sm_2_O_3_ NPs-rGO/PANi	Chemical method;	1.0 × 10^−13^–1.0 × 10^−8^	1.3 × 10^−14^	49
	Drop-casting			
Graphene sheets/PANi/AuNPs	Chemical method;	1.25 × 10^−12^–5 × 10^−8^	2.5 × 10^−13^	50
	Drop-casting			
Graphene sheets-chitosan/PANi/AuNPs	Drop-casting;	1.0 × 10^−11^–1.0 × 10^−9^	2.11 × 10^−12^	8
	Electrochemical method			
ERGO/PANi NWs/AgNFs	Three-step electrochemical method	1.0 × 10^−14^–1.0 × 10^−7^	2.70 × 10^−15^	This work

## 4. Conclusion

The sandwich-structured ERGO/PANi NWs/AgNFs nanocomposite was synthesized directly onto the fabricated Pt microelectrode (0.80 mm^2^ area) using the novel three-step electrochemical approach: electrosynthesis of ERGO, electropolymerization of PANi NWs, and electrodeposition of AgNFs. The obtained ERGO/PANi NWs/AgNFs nanomaterial was characterized by FT-IR, Raman, SEM, EDX, CV and EIS measurements. The optimized ERGO/PANi NWs/AgNFs nanocomposite was used for the first time to develop the electrochemical DNA sensor. The unique properties of the electrosynthesized ERGO/PANi NWs/AgNFs nanocomposite including good adhesion with the electrode surface, large surface area, high electrochemical activity and good biocompatibility made the DNA probe immobilization more simple and effective, and improved remarkably the electrochemical signal of the DNA sensor. The detection limit of the fabricated DNA sensor was just 2.70 × 10^−15^ M. The proposed DNA sensor exhibited outstanding characteristics, such as direct detection, high sensitivity, good specificity and easy miniaturization for fabrication of a lab-on-a-chip system.

## Conflicts of interest

There are no conflicts to declare.

## Supplementary Material
